# The "Excellence in Translational Medicine" and "Bedside-to-Bench" Awards 2007–08

**DOI:** 10.1186/1479-5876-7-57

**Published:** 2009-07-09

**Authors:** Richard J Ablin, Francesco M Marincola, Pier Giorgio Natali

**Affiliations:** 1 Departments of Immunobiology and Pathology, University of Arizona College of Medicine, Arizona Cancer Center and BIO5 Institute, Tucson, AZ 85724, USA; 2 Infectious Disease and Immunogenetics Section (IDIS), Department of Transfusion Medicine, Clinical Center, National Institutes of Health, Bethesda, MD 20892, USA; 3 Immunology and Molecular Pathology Laboratories, "Regina Elena" National Cancer Institute, Rome, Italy

## Editorial

In a continuing endeavor to recognize outstanding contributions in the field of translational medicine, the Editorial Board of the Journal of Translational Medicine (JTM) established "The Excellence in Translational Medicine Award" in 2006 [1]. With the thought to also recognize excellent studies, defined as those exclusively based on the study of human subjects, the Editorial Board has further established "The Bedside-to-Bench Award" in 2008 [2].

The recipient of "The Excellence in Translational Medicine Award" will receive a $4,000 prize, (of which $1,500 is sponsored by *Pfizer Global Research and Development *and* Global Translational Medicine *and $2,500 contributed anonymously). The recipient of "The Bedside-to-Bench Award" will receive $5,000, generously provided anonymously. The funds received from each Award are to be used to cover expenses for any meeting sponsored by a non-for-profit organization that is relevant to the goal of translational medicine and research.

Twenty-two papers (9 self-nominated and 13 highly accessed) from investigators representative of nine countries of four continents, covering a wide range of disciplines published in JTM between 1 July 2007–30 June 2008 were evaluated. For this purpose, an Award Committee* comprised of ten members of the Editorial Board and one non-editorial board member selected and co-chaired by Richard J. Ablin (University of Arizona College of Medicine and the Arizona Cancer Center, Tucson, AZ) and Pier Giorgio Natali ("Regina Elena", National Cancer Institute, Rome, Italy) was formed. The National Institutes of Health Scoring System of 1–5, with 1 = Outstanding and 5 = Poor were used with the papers being evaluated with regard to their:

• Scientific merit

• Originality

• Clarity

• Relevance to the purposes of translational medicine and research (and in "The Bedside-to-Bench Award" to direct study of human subjects)

• Research design

• Methodology

## Excellence in Translational Medicine Award

Given the papers by Ying Jiang [3], Merck Research Laboratories (West Point, PA) and Louise Rodino-Klapac [4], Columbus Children's Research Institute (Columbus, OH), and their respective co-workers were separated in the evaluation by but "0.005" points, they were chosen co-recipients of the "Excellence in Translational Medicine Award" for 2007–08.

With the necessity in drug development to make appropriate and cost effective "go" vs. "no go" decisions based on safety, Jiang et al. [3] demonstrated the use of toxicogenomics for the diagnosis of drug-induced renal proximal tubule toxicity. Their demonstration of the excellent diagnostic performance of the use of genes to identify changes associated with drug-induced toxicities using renal proximal tubule injury as a paradigm provides a basis for the potential translational application of toxicogenomics to reduce the cost of drug development and improve the attrition rates of new chemical entities in drug development.

Corticosteriods, prolonging ambulation, provides a limited, at best treatment option for Duchenne muscular dystrophy (DMD). Multiple treatment options under development include gene replacement therapy. In the report by Rodino-Klapac et al. [4], co-recipient of the "Excellence in Translational Award" for 2007–08, the demonstration and applicability of the regional vascular vs. muscular delivery of recombinant adeno-associated viral vectors carrying a micro-dystrophin cDNA in mice and non-human primates have been evaluated. The study demonstrated, among other criteria, that a regional vascular delivery protects the host from widespread dissemination of virus, and fulfills the necessary criteria for gene delivery with implications for potential clinical application in children with DMD.

## Bedside-to-Bench Award

Regulatory T cells (Tregs; CD4+CD25+Foxp3+), fundamental in maintaining tolerance to self-antigens, can thwart T cell immunity to tumour-associated antigens and thereby, represent a major obstacle to immunotherapy. Thus, reducing their number or inhibiting their effector functions intuitively has the potential of increasing the efficacy of anti-tumour immunity. While increasing preclinical data support this hypothesis, appropriate proof of concept trials in man are yet to be demonstrated.

The contribution of Mary Ann Rasku et al. [5], Graham Brown Cancer Center (Louisville, KY) selected as the recipient of the "Bedside-to-Bench Award" for 2007–08, has addressed this issue in metastatic cutaneous melanoma. This investigator-initiated Phase II Clinical Trial of metastatic melanoma, known for its resistance to treatment, documented that transient depletion of Tregs via administration of an IL-2 immunotoxin, which targets the CD25 marker, is followed by the *de novo *appearance of melanoma antigen-specific CD8+ T cells. By extensively relying on the analysis of patient samples, the study by Rasku et al. [5], represents a sound basis to address unanswered issues in this emerging basic and clinical research area in animal models and man toward delineating, e.g., the relative effects of T cells and depletion of Tregs. In the process, the paper by Rasku et al. [5] exemplifies the journey of "Translational Medicine" between laboratory and the clinic and provides an excellent basis for further studies of T cell depleting agents and their efficacy in cancer patients.

With congratulations to Ying Jiang [3], Louise Rodino-Klapac [4] and Mary Ann Rasku [5] and co-workers, the *2nd *"Excellence in Translational Medicine" and 1^st ^"Bedside-to-Bench" Awards are now history. We are hopeful these Awards will serve to encourage other investigators devoted to improving the "bench-to-bedside" and "bedside-to-bench" concepts of translational medicine and respective initiatives. Competition is now open for the subsequent Awards in each of the two categories in which, we very much look forward to the opportunity of selecting next year's winning papers.

*"Excellence in Translational Medicine and Bedside-to-Bench Awards Committee": Richard J. Ablin (Co-Chairman); Jean-Pierre Armand; Howard L. Kaufman; Bruce Litman; Pier Giorgio Natali (Co-Chairman); Hideho Okada; Michael Perricone; Rja K. Puri; Noriyuki Sato; Patrick F. Terry and Craig Webb.
